# Book Reviews

**Published:** 1805-09-01

**Authors:** 


### CRITICAL ANALYSIS
### OF THE
### RECENT PUBLICATIONS
### ON THE
### DIFFERENT BRANCHES OF PHYSIC, SURGERY, AND MEDICAL PHILOSOPHY.

---

*Salutary Cautions respecting the Gout, in which the Doctrines maintained in a recent Publication, by* Dr. KINGLAKE, *are exposed and refuted, by* JOHN HUNT, *Author of Historical Surgery.*— 8vo. pp. 94, London.

WHEN we were informed, that the author of "Historical Surgery" had engaged in this cold water controversy, we expected an accurate statement of the various periods at which this practice was recommended, from the first records we have of it in the writings of one of the *principes artis medicinæ* to its last renewal under Dr. Kinglake, and his disciples. If we were a little doubtful from the promises contained in the title page, we were not better satisfied after perusing the first paragraph of the preface.

"If (says our Author) apologies for imperfections are under any circumstances admissable, it must be in such cases where the *spur* of the occasion prevents that care and attention which are indispensably necessary for the completo discussion of a subject. But in the present instance the flames of enthusiasm have burst forth, and I fear that all the new invented powers of Refrigeration will not be sufficient to stop their destructive progress. In such alarming situation, delay might be productive of the most important consequences; and the elegancy or energy of an address would not make up for the loss of time, if the salutary caution should unfortunately come too late."

The latter part of this apology is perfectly admissible. Want of elegance or energy may be pardoned.—The first is altogether unnecessary, and we have not found our author at all deficient in the latter; but whilst Dr. Kinglake has so many other antagonists, we cannot see the necessity of so much *spurring*, as to "supercede that care and attention which are necessary for the complete discussion of a subject." As the apology is extended through the whole preface, it may be thought more candid to transcribe it entire. It continues thus:

"A general cry of fire resounds from all parts; but I am apprehensive that water is likely to become the most destructive element on the present occasion. At such a fatal crisis, to remain inactive, or to sit silent in obscurity, correcting an address *(nonumque prematur in annum,)* might be construed into an attempt to eclipse eclipse the fame of Nero, who is said to have amused himself with his fiddle when the capitol was in flames.

"My first object is to sound the alarm, to warn the public of their danger, and protect the podagric multitude from the impending flood. But if the learned doctor, or any of his deluded followers, should presume to oppose this first attack, more circumspection may then be necessary; imperfections may be corrected; the opinions of other authors consulted, and such additional arguments brought forward as the exigency of the occasion may be supposed to require.

"Still I expect the work will not be thought complete; and have no objection to acknowledge, that I at first intended to add a plan of treatment more consistent with what may in some degree be considered the general principles of the present state of the practice of physic, and which I shall now reserve for a second publication. But the quantity of evidence which the learned Doctor has presented to our view has enevitably extended the limits of this part of the investigation; and if the whole of the learned Doctor's extensive correspondence were to have the same degree of attention, it would be impossible to anticipate the bounds. It is not with any intention of avoiding the discussion that so many cases are permitted to pass unnoticed, but it was impossible to do equal justice to the whole; yet if either the Doctor, or any of his fiery advocates, should be inclined to give the challenge, let me now beg leave to assure them, that they may with confidence depend on the most immediate and respectful attention."

The work begins with some pertinent remarks on the uncertainty of physic, and the danger of imputing too much to the fortunate administration of any new remedy, or of confounding the spontaneous operations of nature with the effects of our skill. This introduces some pointed expressions on Dr. Kinglake, whose language, it must be confessed, is, throughout his whole book, so open to sarcasm, that we cannot wonder if Mr. Hunt has availed himself of this advantage over his adversary. Some of his remarks, however, are not only pointed, but so severe, that we shall waive any transcript of them; and only hint to our author, that it is possible his manner of writing may *inflame,* instead of convincing *so fiery a* writer.

Finding a reference to an opinion concerning gouty metastasis, contained in "Historical Surgery," we thought it our duty to turn to the passage. We think it not less our duty to admit, that we were charmed with the bold and not less accurate reasoning with which the passage abounds. But in this, as in every other part, we could not help lamenting the petulence, shall we call it, or unfeeling wantonness, with which the author throws his random shot about him. Without entering into a disquisition of the theory, we shall in general remark, that what is usually called gouty metastatis, is considered as a new disease, which supercedes the gouty action; that this new disease may be treated conformably

 to to its nature, as we should treat it in any other subject; and that when we have subdued it, the gout will probably again resume its action. No one will doubt the ingenuity of this reasoning, which the author supports by his own experience, and by a variety of well authenticated facts, extracted from the writings of others, who have produced them without any view to the theory he has formed.

The foundation, or perhaps we should say, the illustrations of the doctrine, rests on the well known observation of Mr. Hunter, that two diseased actions cannot exist in a part, or in the constitution, at the same time. The analogy, though imperfect, is extremely well preserved; but here we must lament that Mr. Hunt, who always, to do him justice, wishes to pursue the noblest game, cannot introduce Mr. Hunter's name without a bye blow or two.

Mr. Hunter's doctrine of the incompatibility of two actions at the same time is traced as far back as Hippocrates, and again to Horace. Lastly, the illustration of the doctrine, in a case of small-pox and measles, is quoted from an early number of "Medical Commentaries." Yet Hippocrates only says, two pains existing at the same time, the greater will obscure the less. Horace only compares the waverings of the enthusiast with the succession of one disease to another; and Dr. Manget, in the "Commentaries," gives his case rather as a singular occurrence than as a general law. Mr. Hunter might therefore have fairly quoted it as a proof of his doctrine, instead of fearing the recital of it as a means of invalidating his claim to the discovery of the law. It is moreover highly probable that the case would have passed unnoticed, had not Mr. Hunter's opinions been by that time pretty generally diffused among all those who considered medicine as a branch of philosophy, as well as a means of acquiring money. Mr. Hunter began his regular course of lectures a year before the volume alluded to is dated, and a year after the date of the case: for ten years before, he had taught practical anatomy; and all who knew him are well aware of his fondness to communicate whatever he had observed, and his backwardness to publish.

Our readers will excuse this apparent review of one book whilst another is before us; but the reference seemed necessary, and we have explained the consequence.—To return then to the "Salutary Cautions," which we shall dismiss in very few words. We heartily recommend it to the perusal of our readers, and particularly to Dr. Kinglake.

We trust, if nine years be thought too long a silence, nine months may produce a more finished offspring. Of Mr. Hunt's extensive reading we can form no doubt; of his abilities, we are equally well satisfied: but whilst he is constantly on the watch for the errors and imperfections of others, we wish he would suspect a few in his own writingss

---

The

*The Edinburgh Medical and Surgical Journal, exhibiting a concise View of the latest and most important Discoveries in Medicine, Surgery, and Pharmacy. No. 3. July,* 1803.

This Number has reached us with more regularity than either of the former, yet we have no reason to complain that it is inferior in point of merit. As must always be the case in collections of this kind, the original communications are very unequal in value and importance, some however must atone for the deficiencies of others. The first will need no apology.

" *Essay on the Analysis of Animal Fluids, principally with the View of ascertaining their definite Characters.* By John Bostock, M. D. *of Liverpool.*

" The precision," says the author of this paper, "which the analysis of mineral and vegetable substances has obtained, does not appear to be yet extended to the products of the animal kingdom. This remark may be applied both to the solids and fluids which compose the animal body ; but is the most applicable to the latter class of substances. The terms serous, mucilaginous, gelatinous, &c. are employed, even by the most esteemed medical and physiological writers, in a vague and indeterminate manner, without attending either to the original import of the words, or to the restricted meaning which it is necessary to impose upon popular expressions, when they are adopted in scientific researches. The object of the present paper is to ascertain a definite character for what I propose to call the primary animal fluids, and to discover accurate and delicate tests, by which their presence may be easily and certainly indicated. By primary fluids, I mean those into which the compound fluids existing in the animal body are capable of being resolved by the application of different reagents, without decomposing them into their ultimate elements."

The first of the fluids taken notice of is albumen. With the exception of water, no fluid seems to enter so largely into the composition of the animal body. It is said to form a very considerable portion of the blood, and to be found in greater or less quantities in all the secretions. Though it is generally supposed, by the most eminent chemists, that white of egg is composed entirely of this substance, yet by careful and repeated dilution and evaporation it was found to contain in 100 grains, 80 grains of water, 4.5 grains of uncoagulable matter, and 15.5 grains only of pure albumen.

The most distinguishing characteristic of albumen, Dr. Bostock found was the property of being coagulated by heat, which forms an easy test of its existence in any animal fluid. In order to ascertain how small a quantity of albumen could by this means be rendered visible, he added 13 grains of the white of egg to 87 grains of water, thus forming a solution, one grain of which contained $\frac{1}{50}$ of pure albumen. Five grains of this solution were then added to 95 grains of water, so that 100 grains of the fluid contained $\frac{1}{10}$ grain, or $\frac{1}{1000}$ of its weight of pure albumen. This exposed to the heat of of boiling water became perceptibly opake. After this the ingenious author tried the effects of oxymuriate of mercury—of nitro muriate of tin—of tan—of aqua lithargyri acetata—of nitrate of silver—of nitro muriate of gold—and of alum. All these experiments were conducted with the nicest accuracy, and varied according as the attraction of the different tests to any probable combination of the diluting water or surrounding air might produce any uncertainty. To show the manner in which the experiments were conducted, and how much our author was prepared for every possible objection, we shall transcribe his process with alum, and his general remarks on albumen.

"Albumen, in a concentrated state, is powerfully coagulated by alum: I found, however, that this reagent is not so accurate a test of its presence, when in a diluted state, as some of those which I had already employed. One-fifth of a grain of albumen, dissolved in one hundred grains of water, was indeed rendered slightly turbid by the addition of a few drops of a saturated solution of alum; but no precipitate was formed. Before I conclude my account of these experiments, I must observe, that the strength of the solution of albumen was, in all cases, rather less than my estimate. When I added the albumen to the water, a small portion of it always remained insoluble, and this was separated from the fluid by filtration, before the experiments were performed. This insoluble part I supposed to consist of the membranous matter, with which it is said that the white of the egg is intersected. The quantity was indeed almost too small to be appreciated; but where it is desirable to attain as much accuracy as possible, I think it necessary to mention every circumstance which may in the smallest degree affect the result.

"The experiments related above will, I conceive, indicate, with a sufficient degree of accuracy, the presence of albumen as a constituent in an animal fluid. The property of being coagulated by heat is a characteristic of this substance, which will always serve as a mark of discrimination; and we have found that this property is not destroyed by dilution with one thousand times its weight of water. This, therefore, may be considered as a test of its presence, minute enough for all practical purposes. We have also found that there are several reagents which possess the power of precipitating it from its solution in water, while existing only in the same proportion. It will, however, be necessary to observe their operation upon the other animal substances, before we can determine their use in the analysis of compound fluids."

The next subject examined is Jelly, the peculiar property of which, as our author observes, is that of concreting with cold and liquifying in a very gentle heat. It is found to make a small part of the composition of the blood, and a very considerable constituent of membranes, ligaments, cartilages, and tendons. Dr. Bostock chose isinglass for his subject, a substance supposed to contain jelly in its purest state. He found four grains of this dissolved in two hundred hundred of water perfectly concrete by cooling. The temperature of the air is not mentioned, but probably by the expression the solution was somewhat above that degree. When this solution was reduced by double its weight of water, in which the proportion of water to jelly was as one to one hundred, still the mixture stiffened when cold; even with the addition of water so as to reduce the solution to one part of jelly in one hundred and fifty of water, the mixture became mucilaginous though it did not concrete.

The tanning principle proved so nice a test of the presence of jelly, that when to a solution containing only $\frac{1}{5000}$ of its weight in water, a few drops of solution of galls were added, a considerable precipitation followed.

Our author's experiments on mucus were confined to saliva diluted with water and filtered, and to water filtered after an oyster had been for some time agitated therein. Aqua lithargyri acetati in both instances produced a copious precipitation. The following is the result drawn from the whole.

"I apprehend that these experiments will be deemed sufficient to establish a decided and essential difference between mucus and jelly. Independant of the gelatinizing property of the latter, the effects produced upon them by the tanning principle, and by the aqua lithargyri acetati, are exactly opposite. Tan is a most delicate test of jelly, but does not in any degree affect mucus. Aqua lithargyri acetati is a delicate test of mucus, but does not in any degree affect jelly. The oxymuriate of mercury, on the contrary, which is one of the most accurate tests of albumen, does not appear to be affected either by jelly or by mucus.

"Albumen, jelly, and mucus, I am inclined to consider as the only primary fluids which are dispersed through the different parts of the body. Particular vessels or glands contain and secrete particular fluids, which cannot be resolved into other fluids without decomposition, as the fibrine of the blood, the resin of the bile, the urea of the kidney, &c.; but these are, in all instances, confined to their appropriate organs, and do not necessarily enter into the present investigation.

"From the above experiments, I think we may be enabled to lay down, with a considerable degree of accuracy, the leading characteristics of the three primary animal fluids, and to establish tests, by which their presence may be minutely ascertained. The most remarkable property of albumen is its becoming coagulated by heat; a property which it retains so far as to communicate a degree of opacity to its solution in water, when it forms only $\frac{1}{1000}$ part of its weight. A solution of the same strength has its albumen precipitated by the oxymuriate of mercury, and this test will indicate its presence, when composing no more than $\frac{1}{2000}$ of the mixture. The tanning principle, the aqua lithargyri acetati, the nitrate of silver, and the nitro-muriate of gold, are all tests of the presence of albumen, nearly as minute as the oxymuriate of mercury; but they are less valuable, because their effects are not confined to

2 albumen.

albumen. The nitro-muriate of tin and alum are also precipitants of albumen, but they are less delicate in their operation than the reagents enumerated above.

"The peculiar characteristic of jelly is its property of becoming concrete by cold, and being again rendered fluid by a gentle heat; we have found that its solution in water retains this property when it composes $\frac{1}{100}$ part only of the weight of the fluid. Tan is a still more minute test of jelly than of albumen; but jelly is not in the least degree affected by the oxymuriate of mercury, and may thus, in all cases, be easily distinguished from it. No effect is produced in jelly by goulard, and scarcely any by the nitrate of silver, and the nitro-muriate of tin, when it is in a state of much dilution. By means of tan, jelly may be easily detected in a fluid of which it forms only $\frac{1}{5000}$ part.

"The properties of mucus are principally negative; it is not coagulable by heat, nor capable of becoming gelatinized; it is not precipitable either by the oxymuriate of mercury or by tan, but it may be detected with considerable minuteness by the aqua lithargyri acetati.

"It appears, therefore, that the oxymuriate of mercury, tan, and the aqua lithargyri acetati, are the three most valuable tests. The nitro-muriate of tin is a less delicate test of albumen than the oxymuriate of mercury, and is also in some degree affected by jelly. The nitrate of silver appears to be a very nice test of albumen; but it is objectionable, in consequence of its being decomposed by the muriate of soda, a salt which is supposed to exist in most of the animal fluids. The nitro-muriate of gold is a delicate test of albumen, but it likewise precipitates jelly.

"In the analysis of a fluid which is supposed to contain either albumen, jelly, or mucus, the first step is to observe the effect of the oxymuriate of mercury; if this produce no precipitate, we may be certain that the fluid in question contains no albumen. We must next employ the infusion of galls, and if this also cause no precipitate, we may conclude that the animal matter held in solution consists of mucus alone.

"I have before remarked, that the ideas which I have formed of the nature of jelly and mucus, and the relation which these substances bear to each other, differ considerably from those of Mr. Hatchett. It is not, indeed, without a degree of diffidence that I dissent from so distinguished a chemist; but I conceive that I am justified by the experiments related in this essay. Mr. Hatchett, in the valuable paper to which I have already referred, speaks of the white of the egg as consisting of pure albumen; but I believe that, in this particular, he will be found not perfectly accurate.

"There is a great resemblance between the physical properties of animal mucus and vegetable gum; and I found that they strongly resemble each other also in their chemical qualities. A solution of gum arabic, containing one grain of gum to two hundred dred grains of water, was not affected either by the oxymuriate of mercury, or by tan. With the nitro-muriate of tin, and with the nitrate of silver, there was only a slight degree of opacity; but with the aqua lithargyri acetati there was a dense precipitate instantly formed."

Article 2.—"*A Memoir on the State of Health of the* 88*th Regiment, and of the Corps attached to it, from June* 1, 1800, *to May* 31, 1801, *as originally presented to the Medical Board, Bombay; by* James M'Gregor, A. M. M. D. Surgeon to H. M. 88th Regiment and Member of the Royal College of Surgeons, &c. &c."

This paper is very long, and we suppose is such as the Board, to which it was presented, requires of the army-surgeons in that country. It should however have been considerably altered for the collection to which it is here attached. That it contains useful facts and judicious remarks cannot be doubted; but fewer cases, well selected, and related with all their circumstances, detached from extraneous matter, would have been much more desirable. At present the materials are so peculiarly disposed that we have found it difficult to make any selection that could interest or improve our readers.

Article 3.—"*A Case of compound Fracture of the Humerus, from a Gun-Shot Wound, in which Amputation was performed at the Shoulder-Joint; by* R. Watson Robinson, M. D. F. L. S."

This is a valuable case in every respect, and in its relation replete with useful observations. The whole does great credit to the ingenious writer.

Article 4.—"*History of a Case of Trismus, in which the Affusion of cold Water was successfully employed;* by Wm. Dalrymple, *Surgeon, Norwich.*"

This was one of those cases of lock-jaw which will sometimes occur in very irritable habits. We sincerely wish the same remedy may succeed in other instances, but from our own experience cannot encourage the hope.

Article 5.—"*Remarkable Case of Tympanites; by* J. Charles Collins, Surgeon, Swansea, Member of the Royal College of Surgeons of London, and of the Medical Societies of Edinburgh and Guy's Hospital."

This case is very interesting, and in most respects new. It seems difficult to draw any practical inference from it; but we trust the author will not fail to acquaint the public with the result, let it prove what it may.

Article 6.—"*Case of Torpor from Cold, and some general Observations on the Effects of diminished Temperature upon the living System;* by George Kellie, M. D. President of the Royal Medical Society, and Fellow of the Royal College of Surgeons, Edinburgh."

This case is worth recording, inasmuch as it may add to the register of those in which animation has been restored after appearances ances very unfavourable. It is however, as our readers will see, much less remarkable than many others which have been related.

"On the evening of the 26th of February, I was requested by Mr. Charles Cheyne, in the absence of my friend Dr. Cheyne, to visit William Dennis, a lad of 16 years, belonging to the Glasgow Packet, who had been found (as was supposed, dead) in a boat at the end of Leith Pier.

"He had, along with some companions, left the harbour about one o'clock, with the intention of taking a sail in the roads; and on attempting to regain the harbour, between four and five in the evening, the boat grounded on the flats a little to the east of the pier. His companions got ashore, leaving him in charge of the boat, and promising to return to his relief, so soon as they had refreshed themselves.

"Two seamen, who were walking the pier some time after, observed him stretched out in the stern of the boat, as they imagined, asleep; but, becoming alarmed, they went to his assistance about half past seven. He was found cold and insensible, and immediately transported to a neighbouring house, where we soon after saw him. When we arrived, he was stretched out, before the fire, on his back, with very little appearance of life; the whole body, with the exception of the face, which was well coloured, was of a deadly pale appearance, and very cold. The powers of sensation and of muscular motion, were completely suspended. The head and limbs, perfectly flexible, fell lifeless to the ground, from whatever position they were raised to; the mouth was half open, and the jaw, obedient only to the hand, could be moved upwards and downwards, but returned to the half closed position; the respiration was obscure and insensible; but the pulse was quite distinct even at the wrist, though irregular and slow. The organs of sense were equally inexcitable; a candle held close to the exposed eyes made no impression, the eye-balls remained fixed and motionless; the pupils, though dilated, contracted irregularly, while yet exposed to the light, in the way I have sometimes observed them to do in the recently dead."

It is hardly necessary to add any account of the means used for resuscitation, and still less of the author's reflections. We must remark however that he advises an *immediate* application of heat to the whole body, where the actions of the whole are so nearly suspended. This is contrary to the custom in colder climates, the inhabitants of which most frequently meet with such cases.

Article 7.—"*An Account of the Morbid Appearances observed in Two Cases of Diabetes Mellitus*; by DANIEL RUTHERFORD, M. D. Professor of Botany in the University of Edinburgh."

This paper is so valuable, and the cases expressed with so much brevity, compared with their minuteness, that we shall make no apology for transcribing the whole.

"*Dissection*

*"Dissection of John Robinson."*

"Upon opening the body, a much greater quantity of fat was found both under the skin, and in the cavities of the abdomen and thorax, than might have been expected from the apparent emaciation of the body; indeed, the skin was uncommonly thin.

"In the abdomen the blood-vessels all seemed unusually large, and very much distended with blood. The omentum contained a considerable quantity of fat, around large veins filled with black blood. The stomach and small intestines were of natural size and appearance, except that, on the coats of the intestines, the vessels were all very large, and distended with blood, even at their minutest ramifications, giving them exactly the appearance as if they had been well filled with a red injection. The coats of the smaller intestines seemed thicker, and more pulpy than usual. The colon was very large, and distended with air to the utmost; it formed a large doubling under the liver; and throughout its whole length, contained very many little balls of hardened fæces. The mesentery was considerably loaded with fat. The vessels were proportionally large and distended as those of the omentum. The lymphatic glands were uncommonly large, soft, and red; their surface too was painted with many red vessels, or with vessels filled with red blood.

"The liver, perhaps rather small, was otherwise in every respect in the most natural state, except that it lay far up under the edge of the ribs, pressed thither perhaps by the distended colon. The gall bladder rather large and flaccid, yet containing a considerable quantity of very deep yellow bile. The pancreas was perfectly sound and natural, and also the spleen, though perhaps rather large. The kidneys were much augmented in size, soft, with their surfaces painted with numerous vessels. The vessels were uncommonly dilated. The emulgent vein, *e. g.* of the right side, was not under three-fourths of an inch in diameter, as it appeared distended with blood. The ureters also were considerably dilated, the bladder seemed capacious, its coats thickened; and it was half filled with liquid.

"In the thorax there was a considerable quantity of fat in the mediastinum, and in the lower part especially, running along the diaphragm. The lungs were apparently perfectly sound; a few adhesions were observed betwixt them and the sides of the thorax, and also betwixt them and the pericardium. The lungs in texture were perfectly right, or even perhaps more spongy and pervious to air than common. The heart of moderate size, flaccid, and pale, though the coronary veins were greatly distended with black blood. The large cavities contained a considerable quantity of very dark blood, generally fluid, though some had coagulated slightly both in the right auricle and ventricle. In the anterior mediastinum, in the site of the thymus, was a cluster of glands altogether like those of the mesentery, in several of which were calculous concretions, one not much smaller than a small horse bean. The proper glands glands of the lungs, those situated at the ramifications of the trachea, were unusually large, soft, and of a brilliant black colour.

The age of this man was between forty and fifty.

" *Dissection of Ann Laidlaw, aged nine or ten.*

" On external examination, every part of the body seemed to be much emaciated and reduced in bulk, except the abdomen, which was uncommonly distended with what was afterwards found to be air in the stomach and smaller intestines, and fæces collected in the larger ones.

" When the muscles covering the abdomen and thorax were thrown back, they seemed to be very slender, and of a rather more florid red colour than they usually are in the healthy subject. The fat was entirely abolished, nothing being left but the cells in which it was formerly contained. The lungs on both sides adhered slightly in some places to the pleura costales; but, on the right side, besides adhering every where very firmly to the pleura of the mediastinum, a quantity of coagulable lymph, of a yellowish colour, was found effused between the pleura of the inferior and middle lobes of the lungs and the mediastinum. On both sides, the lungs were rather of a pale colour externally, but they felt firmer to the touch than in the healthy state; and, on cutting into the substance of them, a puriform fluid was seen exuding from different places, although no distinct tubercles could be perceived, and there was little more than the common quantity of serum effused into either cavity of the thorax.

The pericardium contained about four ounces of a yellowish transparent serum. The heart was of the natural size, unusually free from fat, and rather of a pale colour. The right ventricle very flaccid, the left firm and contracted. The internal structure of the heart was natural; but, on opening the aorta, the inner substance of the anterior side of it was of a red colour, and the redness extended from the commencement of the aorta to that part where it passes through the diaphragm into the abdomen. The aorta below the diaphragm was, in every respect, in a healthy state.

" The pulmonary veins and vena cava did not appear to be any way diseased; several of the bronchial glands were considerably enlarged; in particular, one on the right side, which resembled a middling sized walnut, and contained a good deal of a brownish substance, of a consistence rather thinner than cheese. The trachea was perfectly natural.

" The stomach was much distended with air, but, in other respects, had a healthy appearance, both externally, and when it was laid open to examine the internal coat. The upper part of the intestinal canal was uncommonly distended with air, and, in some places, the blood-vessels near the juncture of the mesentery were filled with blood, giving somewhat of the appearance of inflammation. The caput cœcum, the colon, and rectum, were every where filled with hard fæces, by which they were distended to a size size far greater than could have been thought possible in so young a subject; in other respects they were healthy. The glands of the mesentery and mesocolon were enlarged; but, though very numerous, none of them had attained a size larger than a common bean, somewhat flattened, and some of them nearly white. The omentum was small, contracted, and completely free from fat. The kidneys were somewhat enlarged, the colour natural; but, on cutting into them, the *tubuli uriniferi* were fully more distinct than usual, and the pelvis of each was also considerably enlarged; no other morbid change of structure was perceived. The ureters were much larger than common, in some places being nearly half an inch, in other places more, in diameter. The right lobe of the liver adhered firmly to the diaphragm in many places; but, in other respects, both as to external appearance and internal structure, it was quite sound.

" The gall bladder contained a small quantity of light-coloured yellowish bile. The bladder was nearly empty, but of a natural appearance. The spleen, pancreas, uterus, Fallopian tubes, and ovaria, did not seem to be any way diseased."

Article 8.—" *Case of Protrusion of the Tongue.* By W. R. Clanny, M.D. Bishop-Wearmouth."

This is a good practical illustration of a paper given in our Journal, vol. vi. p. 353.

Article 9.—" *Flatulent Colick, with Hernia, cured by Opiates alone, freely administered.* By James Ross, M.D. Montrose."

The subject of this article has a hernia for which he wears an elastic truss. He is also occasionally attacked with flatulent colic, at which time the whole abdomen swells, and a protrusion of the size of a hen's egg appears at the ring. The author of the paper tells us that the omentum, and part of the intestine form the contents of the tumour. Such may be the state of the parts, but we cannot help being surprized at the confidence with which Dr. R. assures us of a thing which would be guest at with some caution by men of the largest opportunities of examining such cases. The protrusion being only the temporary effect of general intumescence, without stricture or strangulation, it is not surprizing that it should so readily yield to opiates and aromatics. However, the narration may be useful in teaching the young practitioner, that under such circumstances evacuation of the bowels is not necessary before the patient is relieved from his immediate sufferings.

Article 10, presents us with a second paper from the Enquirer, " *On the Inexpediency of erecting Foundling Hospitals.*"

This is more properly a political than medical enquiry. However, it is connected with medicine, and the appropriation of a charitable fund towards the erection of such a hospital in Edinburgh, renders the enquiry peculiarly proper at this time. The author, after giving an account of the different institutions for Foundlings throughout the world, has no difficulty in shewing the inutility and danger of such establishments. Mr. Malthus has convinced convinced many sceptics of the same. It is not difficult to show the inconveniences of any thing, and it is far from our intention to engage in so important a controversy: But to all questions there are two sides. We would ask, then, has not the severity of the Scotch kirk hitherto more than justified "*the pious and charitable* purposes of preventing child-murder, of receiving privately women big with child, assisting them in their delivery so as to conceal their shame, and of taking care of their children as foundlings?"

Such are all the original papers. There is indeed another, which we may perhaps hereafter transcribe from the New York Medical Repository, with our doubts and enquiries. It is not *our* intention to accuse Dr. Rush of deserting the sect of Contagionists and embracing an opinion contrary to European belief. For our own parts (perhaps from some unfortunate *mauvaise honte*) we should call the Professor's desertion, as it is here styled, an ingenuous acknowledgement of his former error; and in a disease (the yellow fever) if not altogether trans-atlantic, at least trans-marine, we should not presume to offer more than enquiries to those who have so much larger opportunities of judging than ourselves.

The Postscript contains a valuable communication from Prof. Pacchioni, of Pisa, on the composition of muriatic acid. The whole process is not yet sufficiently detailed, but the general result of his experiments go to prove that,

"1. *Muriatic acid is an oxyd of hydrogen*, and consequently composed of hydrogen and oxygen.

"2. *In the oxygenated muriatic acid, and therefore*, a fortiori, *in muriatic acid, there is much less proportion of oxygen than in water.*

"3. *Hydrogen is susceptable of very many and different degrees of oxydation*, contrary to what is universally believed by pneumatic chemists, who assert that hydrogen is susceptible of only one invariable degree of oxydation, that in which it forms water."

These discoveries were the effects of different attempts to decompose water by the Galvanic pile. Mr. Peel, of Cambridge, our readers must be aware, has been engaged in similar experiments; and we may consider the confirmation of these important facts as a new link to connect that chain of elementary decomposition, for which we are indebted to the honest industry of a Priestly, the perseverance of a Cavendish, and to the original genius of both.

This number contains an engraving to illustrate the valuable communication of Mr. Cooper, contained in the last.

---

*A Clinical History of Diseases. Part First: being,* 1. *A Clinical History of the Acute Rheumatism.* 2 *A Clinical History of the Nodosity of the Joints. By* John Haygarth, M. D. F. R. S. Edin. &c.

Of the value of faithful records of medical practice, given by men of mature judgment and large experience, and free from every bias of system, no one who is himself a practitioner can entertain any any doubt; and much it is to be regretted that the veterans of the profession are generally too fond of the ease they have purchased by their labours, and too indifferent to the instruction of their successors, to take the trouble of methodising the important facts which must have passed under their notice.

The late Dr. Heberden was a laudable exception to this charge, and merited the grateful acknowledgements of his brethren by his valuable legacy of observations.

Dr. Haygarth has too well established his character for benevolent ardour in promoting the welfare of society, and for assiduous attention to the improvement of his profession, to occasion any surprise that, while still engaged in active duties, he has followed such a respectable example. He has given us reason to hope, that the present publication is only the forerunner of a series of clinical histories, which will comprize every thing of importance that has occurred in an extensive practice of thirty-five years, carefully noted down at the time; and from the present specimen we cannot doubt that much benefit will be the result.

The history of the Acute Rheumatism is arranged in two sections, of which, the first contains a plain and popular account of the causes, symptoms, and mode of cure; the second consists of proofs and illustrations, referring particularly to actual cases in the author's own practice. Of these last there are 170, concisely disposed in a tabular form, under a variety of heads, which exhibit at one view the leading circumstances and the remedies employed. The practical point chiefly inculcated in this clinical history, is the early exhibition of the Peruvian bark; that is, without waiting for the subsidence of the fever or inflammation, and only premising evacuation of the primæ viæ by antimonials. This practice the author derived from the late Dr. Fothergill, who informed him of his having been instructed in it by Sir Edward Hulse; and from that physician, it is traced to Morton in his Treatise on Fevers. Dr. Haygarth gives a very exact statement of the time of the disease, and mode in which he has employed this remedy, and the results; and very fairly discusses the question, whether in the unfavourables cases it did harm. We cannot doubt that this testimony will be thought very conclusive on this head, and will go far to establish the practice. We shall copy two of the cases, which are narrated at length, as examples of the successful exhibition of the bark.

" No. 134. Mr. W. July 13, 1791, having caught cold, has been ill for five, especially for the last two weeks. The joints of his fingers, feet, shoulders, hands are swelled, red and painful. Profuse sweats, flatulence; much rumbling in his bowels; in a laxative state; shortness of breath; faintness; tears; pulse 96.

" He has been bled seven times from the arm; the last blood taken to-day has an inflammatory crust.

" At first x and soon xx grains of the powder of bark were taken in mint water every three hours.

"July 21. He has taken four pints of the mixture, which contained 5 $\frac{1}{6}$ ounces of bark in substance, in eight days. As soon as he began the medicine, there was an immediate abatement of the inflammation, flatulence, and languor. In five days his sweats ceased; in six days after he began to take the bark he was so well recovered as to ride on horse-back. No complaint remained, but some pain of one hand and one shoulder.

"In this case the efficacy of the bark was remarkable. Perhaps the frequent blood-letting, which had been previously employed might be conducive to this purpose. However, it is manifest that such copious evacuations from his veins did not cure the rheumatic inflammation, but reduced the patient to extreme languor, debility, and even tears.

"No. 170. Mrs. M. March 28, 1803, being the fourth day of a rheumatic fever, had suffered chills, burnings, profuse sweats, violent pain of the shoulders, back, elbows, feet, thigh, knee, hips; great thirst; very restless days and nights. She took the antimonial powder, which produced copious evacuations of her stomach and bowels, but without relief of the fever or inflammation. March 29. Bark in powder gr. x. was given every third hour. March 30. The pain of the shoulders remained, but it was diminished in the lower extremities; less sweat. The bark feels grateful. A cough. On account of this symptom, the bark was omitted, and the antimonial powders repeated. March 30, 31, April 1 and 2, without producing any abatement of the pains, swellings, or fever. I endeavoured repeatedly to persuade this patient to lose blood by the lancet, chiefly on account of her cough, but could not overcome her prejudices on this point. She was extremely averse to the operation, and her faint debilitated state gave her apprehensions that she could not bear such an evacuation.

"April 3. The bark was again taken in powder, gr. x. every fourth hour; April 4th, every third hour. April 6. A remarkable abatement of all the symptoms, except the inflammation of the left hand. The bark was increased to gr. xv. every third hour; April 8, to gr. xxii. every third hour. April 10. The pains, swellings, and sweats are much diminished. An ounce and half of the decoction and 20 grains of the powder of bark were then given every three hours. During this course, to alleviate the fever and watchful nights, effervescing draughts, and occasionally with anodynes were administered. April 12. Seldom coughs. Convalescent. Continue bark.

"In fourteen days (April 3 to 17) though the patient in that period thought her pains and fever several times aggravated by catching cold, she was restored to perfect recovery from rheumatism, which had reduced her to the wretched state above described. I never witnessed a more distressing case of this painful malady where the inflammation remained in its usual seat, the joints and muscles, and was not translated upon any of the vital vital organs. By continuing this remedy, her appetite, strength, and sleep returned. She soon again enjoyed the blessings of good health."

The Clinical History of the Nodosity of the Joints, is a brief account of a painful affection which has usually been confounded with gout or rheumatism, but which appears to this writer to be a peculiar disease. It almost exclusively belongs to the female sex, and generally makes its appearance about the period of the cessation of the menses. It attacks a variety of joints, but especially those of the hands and fingers, and produces a gradual enlargement of the ends of the bones, the periosteum, and articular membranes and ligaments, with pain and impeded motion. The remedies found most effectual have been warm bathings, the douche, and leeches.

Dr. Haygarth has not given his cases of this disease in the same tabular form with those of rheumatism, (a mode, which, from the difficulty in drawing up and printing, he is inclined hereafter to disuse) but has formed general results from them, indicating the principal occurrences. It is scarcely necessary for us to recommend to our readers a volume so replete with practical instruction from so respectable a source.

